# De-Escalating and Discontinuing Immunotherapies in Patients With NMOSD and MOGAD

**DOI:** 10.1212/WNL.0000000000218482

**Published:** 2026-08-26

**Authors:** Yael Hacohen, Géraldine Androdias, Georgina Arrambide, Arlette Laurien Bruijstens, Olga Ciccarelli, Alvaro Cobo-Calvo, Eoin P. Flanagan, Ho Jin Kim, Ellen Iacobaeus, Jan D. Lünemann, Bruno Stankoff, Mar Tintore, Romain Marignier

**Affiliations:** 1 Queen Square MS Centre, Department of Neuroinflammation, UCL Queen Square Institute of Neurology, Faculty of Brain Sciences, University College London, United Kingdom;; 2 Pediatric Neurology, Great Ormond Street Hospital for Children, London, United Kingdom;; 3 Service de neurologie, sclérose en plaques, pathologies de la myéline et neuro-inflammation, Centre de Ressources, Recherche et Compétence sur la Sclérose en Plaques, Hôpital Neurologique Pierre Wertheimer, Hospices Civils de Lyon, Lyon-Bron, France;; 4 Clinique de la Sauvegarde, Ramsay Santé, Lyon, France;; 5 Neurology-Neuroimmunology Department, Multiple Sclerosis Centre of Catalonia (Cemcat), Vall d’Hebron Barcelona Hospital Campus, Universitat Autònoma de Barcelona, Spain;; 6 Department of Neurology, Erasmus Medical Center, Rotterdam, the Netherlands;; 7 Faculty of Brain Sciences, University College London, United Kingdom;; 8 National Institute for Health and Care Research (NIHR) University College London Hospitals (UCLH) Biomedical Research Centre, London, United Kingdom;; 9 Department of Neurology and Mayo Clinic Center for Multiple Sclerosis and Autoimmune Neurology, Mayo Clinic, Rochester, MN;; 10 Department of Neurology, National Cancer Center, Goyang, South Korea;; 11 Division of Neurology, Department of Medicine, University of British Columbia, Vancouver, BC, Canada;; 12 Djavad Mowafaghian Centre for Brain Health, University of British Columbia, Vancouver, BC, Canada;; 13 School of Biomedical Engineering, University of British Columbia, Vancouver, BC, Canada;; 14 Department of Neurology with Institute of Translational Neurology, University and University Hospital Münster, Germany;; 15 Department of Neurology, Multiple Sclerosis Center, Pitié-Salpêtrière Hospital, AP-HP, Paris, France;; 16 Sorbonne Université, Paris Brain Institute, ICM, Inserm, CNRS, Hôpital de la Pitié Salpêtrière AP-HP, Paris, France; and; 17 Centre de Référence des Maladies Inflammatoires Rares du Cerveau et de la Moelle, Hôpital Neurologique Pierre Wertheimer, Hospices Civils de Lyon, Lyon-Bron, France.

## Abstract

Antibody-mediated inflammatory diseases of the CNS, including aquaporin-4 antibody neuromyelitis optica spectrum disorder (AQP4-Ab NMOSD) and myelin oligodendrocyte glycoprotein antibody–associated disease (MOGAD), have undergone major therapeutic transformation with the advent of sensitive antibody assays and targeted immunotherapies. These advances have markedly reduced relapse rates and improved long-term outcomes. With improved disease control, questions regarding optimal treatment duration and the possibility of de-escalation or discontinuation are increasingly relevant. In AQP4-Ab NMOSD, relapses are typically severe and disabling, and most observational studies show a high risk of reactivation after tapering or withdrawal. Thus, discontinuation is never recommended. Successful de-escalation has been reported in selected patients with prolonged remission, although relapse risk persists, underscoring the need for individualized decisions and close monitoring. In contrast, MOGAD is clinically heterogeneous. Many patients, particularly children, experience a monophasic illness with good recovery, whereas relapse risk in adults appears to decline after several years. De-escalation strategies can thus be applied for anti-CD20 and IVIG. Recent cohort studies suggest that even discontinuation may be feasible after 2 to 5 years of remission in children and adults, especially in those who become seronegative for myelin oligodendrocyte glycoprotein immunoglobulin G. In seronegative NMOSD, the evidence base is limited and prognosis uncertain; attacks can be severe, no therapies are specifically approved, and although some experts suggest that discontinuation may be considered after 5 years of stability, this remains guided by expert opinion alone. Treatment de-escalation is sometimes necessary because of adverse effects, comorbidities, infections, treatment fatigue, or life circumstances such as pregnancy. In pregnancy, management requires balancing maternal disease control with fetal safety, with strategies ranging from continuation of selected therapies to temporary tapering or deferral. Monitoring with MRI, OCT, and emerging fluid biomarkers offers potential to detect early recurrence of disease activity, although none are validated for routine use. Prospective studies, real-world registry data, and dedicated trials are urgently needed to inform safe and patient-centered de-escalation/discontinuation strategies.

## Introduction

Myelin oligodendrocyte glycoprotein antibody–associated disease (MOGAD) and aquaporin-4 antibody positive neuromyelitis optica spectrum disorder (AQP4-Ab NMOSD) are distinct autoimmune inflammatory disorders of the CNS.^1^ Both conditions are characterized by episodes of optic neuritis, transverse myelitis, brain and brainstem/cerebellar involvement, which can lead to substantial and potentially irreversible neurologic disability. Advances in diagnostic characterization, particularly through the development of cell-based assays for AQP4 and MOG antibodies (Abs), have improved the ability to accurately classify these disorders. This, in turn, has enabled the development of more targeted and effective immunotherapies, thereby reduced relapses, and improved long-term outcomes.^1^ Since the characterization of these 2 antibodies, it has become increasingly clear that AQP4-Ab NMOSD and MOGAD represent truly distinct diseases, with different diagnostic criteria, treatment strategies, and, as will be discussed, different approaches to stopping and de-escalation of treatment.

With improved disease control has come the recognition of new challenges in long-term disease management, including the question of treatment duration. Although the rationale for initiating immunosuppressive therapy after the first attack is clear in AQP4-Ab NMOSD,^1^ the optimal duration of treatment in both antibody-associated disorders remains uncertain. Long-term immunotherapy reduces relapse risk but carries cumulative burdens related to side effects, treatment fatigue/acceptability, costs, and effects on quality of life. It is likely that some patient may require immunosuppressant therapies for decades, which may lead to toxicities, including increased risk of malignancy, acquired immunodeficiencies such as hypogammaglobulinemia and lymphopenia, which, in turn, is likely to increase the risk of infections. In this context, there is growing interest in the possibility of treatment de-escalation or discontinuation in selected patients, after a period of disease stability. This interest is particularly pronounced in MOGAD, where disease course is more variable, and a substantial proportion of patients, especially children, experience a monophasic presentation. In addition, in adults, the risk of relapse tends to reduce after 2 years and becoming very low after 5 years.^2^

Despite the clinical importance of this topic, current guidance on treatment de-escalation and discontinuation is limited. Most available data come from small retrospective cohorts, and there are no prospective, randomized studies specifically designed to evaluate de-escalation and discontinuation strategies in either MOGAD or AQP4-Ab NMOSD. This has resulted in highly variable practices among clinicians and uncertainty for patients.

Here, we review and assess the current evidence, make clinical considerations, and propose future directions for treatment de-escalation and discontinuation in MOGAD and AQP4-Ab NMOSD, including specific consideration in pediatric and pregnancy, incorporating perspectives from patients and global health contexts. We also briefly address double-seronegative NMOSD, particularly considering evolving diagnostic criteria.

This review emerged from an ECTRIMS Workshop held in 2023 in Lisbon on de-escalation strategies in multiple sclerosis (MS) and related disorders. The data regarding MS discussed during the meeting were published in 2025.^3^ We performed a narrative synthesis of the current literature on treatment de-escalation and discontinuation in AQP4-Ab NMOSD and MOGAD. Relevant studies were identified through targeted searches of PubMed and EMBASE, focusing on treatment withdrawal, tapering, dose reduction, and relapse risk. Given the limited number of dedicated discontinuation studies, we also incorporated indirect evidence from observational cohorts and expert clinical experience. This review was not conducted as a formal systematic review or Delphi consensus process but reflects multidisciplinary expert interpretation of the available data.

## De-Escalation and Discontinuation: General Concept and Lessons From MS

Treatment de-escalation refers to the intentional reduction in the intensity of immunosuppressive or immunomodulatory therapy, such as switching to a less potent agent, lowering the dose, or extending dosing intervals, once disease control has been achieved or when treatment side effects shift the benefit-risk balance. Temporary treatment interruptions, such as those related to pregnancy or intercurrent illness, should be distinguished from permanent discontinuation, as the risk profile and clinical management may differ. In contrast, treatment discontinuation describes the complete cessation of therapy.^3^

Those approaches aim to optimize the long-term benefit-risk profile by reducing adverse effects, improving quality of life, and lowering costs, without compromising disease outcomes. Originally developed in oncology, the concept of therapeutic de-escalation has since expanded to a wide range of conditions, including infectious diseases, transplantation, endocrinology, and chronic inflammatory disorders. Although de-escalation and discontinuation share a common rationale and overlapping challenges in patient selection and monitoring, complete treatment cessation is not feasible for all diseases or therapies. A prerequisite for those strategies is evidence of disease stability, determined through clinical, radiologic, and sometimes biological measures. However, reliable biomarkers to guide decision making remain limited in routine practice. As a result, treatment decisions must be individualized, carefully balancing the risks of relapse, rebound, or disease progression against the potential benefits of reducing therapy. Given the lack of universally validated algorithms, shared decision making with patients is essential. Treatment interruption, for example in the context of intercurrent illness or planned pregnancy, introduces further complexity and requires careful monitoring.

In MS, there are accumulative evidence and data supporting the rationale for de-escalation and discontinuation. With advancing age and disease duration, inflammatory activity declines, as reflected by reduced relapse rates and fewer gadolinium-enhancing lesions on MRI.^4^ This change is thought to reflect the effects of immunosenescence and “inflammaging.”^3^ At the same time, the safety profile of disease-modifying therapies (DMTs) becomes less favorable with increasing susceptibility to infections, malignancies, and comorbidities. These factors, supported by observational data and more recently by a randomized controlled trial, suggest that patients aged over 55–60 years with at least 5 years of stable disease may be appropriate candidates for de-escalation or discontinuation.^5,6^ This paradigm cannot be directly translated to AQP4-Ab NMOSD or MOGAD, where pathophysiology, inflammatory burden, and tissue injury differ substantially.

## Treatment De-Escalation and Discontinuation in AQP4-Ab NMOSD: Immunopathogenesis, Evidence, and Clinical Considerations

The immunopathogenesis of AQP4-Ab NMOSD directly informs treatment strategy. AQP4-Ab are directly pathogenic IgG1 autoantibodies, highlighting the central role of antibody-secreting cells and B and T lymphocyte interactions.^1^ Upon target binding, complement activation triggers astrocyte destruction, myelin damage, and secondary axonal loss, with neutrophil, macrophage, and eosinophil infiltration mediated by the membrane attack complex. AQP4-Ab–induced astrocyte activation drives C1q release and IL-6–mediated neuronal damage, whereas microglial activation sustains a chronic inflammatory state requiring ongoing immunotherapy.^1^ Accordingly, B-cell–depleting agents (inebilizumab, rituximab), IL-6 pathway inhibitors (tocilizumab, satralizumab), and complement inhibitors (eculizumab, ravulizumab) have all demonstrated reduced relapse rates and disability prevention.

De-escalation has been evaluated in several studies. Mitoxantrone induction resulted in disease reactivation upon switching to a less potent drug.^7^ In a Chinese cohort of 145 patients followed for a median of 6 years, 20% in remission for over 3 years successfully tapered immunosuppression without relapse, predominantly those with lower baseline Expanded Disability Status Scale (EDSS; median 1.5); conversely, 35% of patients with EDSS ≥3 relapsed after de-escalation.^8^ Rituximab de-escalation by dose reduction^9^ or interval extension^10^ carries relapse rates of 22% and 24%, respectively. In a cohort of 65 patients undergoing 137 rituximab de-escalations, relapse risk was 3.1% after ≥1 year of stability vs 16.1% if earlier, with no relapses in those with fewer than 4 prior attacks attacks.^11^

Discontinuation is generally not recommended given the persistent relapse and disability risk. In a South Korean study, 12/17 patients discontinuing azathioprine or mycophenolate mofetil (MMF) relapsed at a median of 4 months, whereas relapses after rituximab discontinuation occurred at 54 and 85 months.^12^ Severe disease reactivation has also been reported after cessation or switching of complement inhibitors.^13^

### Key Recommendation

In AQP4-Ab NMOSD, treatment discontinuation is not recommended given the persistent risk of severe relapse; de-escalation may be cautiously considered in selected patients with prolonged remission, low disability burden, and fewer prior attacks but requires individualized decision making and close monitoring.

## Treatment De-Escalation and Discontinuation in MOGAD: Immunopathogenesis, Evidence, and Clinical Considerations

MOGAD immunopathogenesis is distinct from AQP4-Ab NMOSD. MOG-IgG1 antibodies activate complement, although deposition is variable and lesion-dependent, with less consistent involvement than in AQP4-Ab NMOSD.^14^ CD4^+^ T-cell–mediated inflammation is also central, with MOG-Ab–driven opsonization facilitating MHC class II antigen presentation and MOG-specific T-cell activation, accounting for the characteristic CD4^+^ T-cell infiltration in MOGAD lesions.^15^ The disease course varies widely.^16^ Children frequently present with postinfectious or postvaccination monophasic diseases, and even relapsing MOGAD relapses less frequently and with more variable interattack intervals than AQP4-Ab NMOSD with generally better recovery and less cumulative disability.^17,18^ Early intervention with corticosteroids or plasma exchange may favorably alter the disease course: in a South Korean study of 240 adults, treatment within 5 days of onset was associated with lower relapse rates and higher seroconversion rates, whereas delay beyond 14 days conferred an almost three-fold higher relapse risk.^19^ A multicenter Italian pediatric study of 75 patients similarly showed that corticosteroids within 7 days reduced the likelihood of a relapsing.^20^ Multiple empiric attack-prevention treatments have been used in MOGAD including azathioprine, MMF, rituximab, intravenous immunoglobulins (IVIg), and tocilizumab.^21-23^ A meta-analysis suggested that relapse-free rates were the highest with IVIg (79%) and tocilizumab (93%).^24^ IVIg is conventionally administered at 1–2 g/kg every 4 weeks,^21^ with treatment de-escalation implemented by reducing the dose and/or prolonging infusion intervals after disease stabilization. Trials of rozanolixizumab, satralizumab, rituximab, azathioprine, and tocilizumab are ongoing.

Regarding discontinuation, a South Korean multicenter cohort of 333 patients^25^ analyzed 41 who discontinued maintenance therapy; 10/41 (24.4%) relapsed at a median of 8.2 months (IQR 6.3–11.5), all in those with prior relapsing disease (10/21; 47.6%), with no relapses in monophasic patients (0/20). Shorter treatment duration predicted relapse (median 9.4 vs 50.9 months, *p* = 0.036). A large UK cohort of 190 patients contributing 236 discontinuation intervals^26^ reported postdiscontinuation relapse in 92/236 cases (39.0%) at a median of 5.4 months (IQR 1.4–20.1), more frequently in relapsing (43/86; 50.0%) than monophasic patients (49/150; 32.7%), with relapsing disease independently predicting relapse (HR 1.95, 95% CI 1.25–3.06). Modeling suggested optimal minimum treatment durations of 10–18 months after a first attack and 20–30 months in relapsing disease. The NOMADMUS French registry^27^ evaluated 83 planned discontinuations (60 oral immunosuppressants, 23 rituximab) in 705 screened patients, of whom 31/83 had relapsing disease at withdrawal; 7/83 relapsed at a median of 0.5 years (IQR 0.1–1.4), with cumulative 1-year relapse incidence of 8.7% (95% CI 1.0%–15.9%). Shorter treatment duration (<1 year) and recent relapse (<2 years prior) predicted relapse, with no rebound activity and minimal disability worsening (median EDSS change 0).

### Key Recommendation

In MOGAD, treatment discontinuation may be cautiously considered in selected patients, particularly those with a monophasic course and prolonged remission, but carries a meaningful relapse risk even in monophasic disease; relapsing disease course, shorter treatment duration, and recent relapse activity are the strongest predictors of postdiscontinuation relapse, requiring individualized decision making and close monitoring, particularly in the first year after withdrawal.

## Seronegative NMOSD

Double-antibody negative NMOSD (seronegative for both AQP4-Ab and MOG-Ab) is considered a pathogenetically heterogeneous condition. As such, most findings related to this subgroup should be interpreted with caution. Currently, no therapies are specifically approved for these patients, and treatment decisions are guided by expert opinion. Standard approaches typically involve classical immunosuppressants or rituximab.

Attacks in seronegative NMOSD can be severe and often result in incomplete recovery.^28^ However, the long-term risk of relapse and disability remains uncertain, with conflicting evidence in the literature.^29^ Evidence on treatment discontinuation in this group is limited. A French nationwide study evaluating rituximab de-escalation in seronegative patients reported an increased risk of attacks within 12 months after dose reduction or discontinuation.^11^ Given the likely pathobiological heterogeneity and variable prognosis of double-antibody negative NMOSD, some experts have proposed that treatment discontinuation could be considered in stable patients, but only after a minimum follow-up period of 5 years.^30^

### Key Recommendation

In seronegative NMOSD, treatment discontinuation is not routinely recommended given the severity of attacks and uncertain prognosis; where considered, it should only be contemplated after a minimum of 5 years of stability, guided by individualized expert opinion and close monitoring.

A structured overview of maintenance therapies including mechanism, efficacy, risks, and cost considerations to contextualize de-escalation/discontinuation decisions are summarized in Table 1.

**Table 1:** Overview of Maintenance Therapies, Including Mechanism, Efficacy, Risks, and Cost Considerations

Class/therapy	Mechanism of action	Evidence for relapse prevention	Key risks/safety issues	Monitoring burden	Relative cost	Practical de-escalation considerations
Anti-CD20: rituximab	B-cell depletion	High efficacy in AQP4-NMOSD (class I evidence); variable in MOGAD	Infusion-reaction, infections, hypogammaglobulinemia, impaired vaccine response	B cells, IgG levels	Low	Interval extension or dose reduction feasible; cessation associated with high relapse risk in NMOSD
Anti-CD19: inebilizumab	B-cell depletion	High efficacy in AQP4-NMOSD (class I evidence); unknown in MOGAD	Infections, hypogammaglobulinemia, impaired vaccine response No data on pregnancy	B cells, IgG levels	High	No data on optimization; cessation associated with high relapse risk in NMOSD
IL-6 inhibitors: tocilizumab	IL-6 receptor blockade	High efficacy in NMOSD (class I evidence); observational data suggest good effectiveness in MOGAD	Infection risk, neutropenia, lipid abnormalities	Blood counts, LFTs	Low	Limited discontinuation data; taper strategies undefined
IL-6 inhibitors: satralizumab	IL-6 receptor blockade	High efficacy in NMOSD and MOGAD (class I evidence)	Infection risk, neutropenia, lipid abnormalities No data on pregnancy	Blood counts, LFTs	High	Limited discontinuation data; taper strategies undefined; delay of efficacy if switch is considered
Complement inhibitors: eculizumab, ravulizumab	Terminal complement blockade	Near-complete relapse suppression in NMOSD (class I evidence); unknown in MOGAD	Meningococcal infection risk, vaccination required, antibiotic prophylaxis recommended	Infection surveillance	High	Stopping associated with severe rebound in case reports
IVIg	Immunomodulatory (Fc receptor, complement, cytokine modulation)	Observational data suggest good effectiveness in MOGAD	Headache, thrombosis (rare), infusion burden	Minimal laboratory monitoring	Moderate	Infusion spacing commonly used as de-escalation strategy
Azathioprine	Purine synthesis inhibition	Observational data suggest moderate effectiveness in NMOSD/MOGAD	Cytopenias, hepatotoxicity, malignancy risk after long-term exposure	Blood counts, LFTs	Low	Typically stopped rather than tapered
MMF	Lymphocyte proliferation inhibition	Observational data suggest moderate effectiveness NMOSD/MOGAD	Gastro-intestinal toxicity, infections, teratogenicity	Blood counts, LFTs	Moderate	Typically stopped rather than tapered
Oral corticosteroids	Broad immunosuppression	Observational data suggest relapse reduction in MOGAD; limited data in NMOSD	Metabolic, bone, psychiatric effects Effect on fetus during first trimester of exposure	Metabolic monitoring	Low	Gradual taper standard; long-term toxicity limits use

Abbreviations: AQP4-Ab = aquaporin-4 antibody; IgG = immunoglobulin G; IVIg = intravenous immunoglobulin; LFT = liver function test; MMF = mycophenolate mofetil; MOGAD = myelin oligodendrocyte glycoprotein antibody–associated disease; NMOSD = neuromyelitis optica spectrum disorder.

## Specific Scenarios

### Pediatrics

Immunosuppressive therapy in children with AQP4-Ab NMOSD or MOGAD requires caution because of the developing immune system, interference with vaccination schedules, and the potential for treatment-related toxicities to affect growth, cognition, and psychosocial development.^31^ Yet, despite these concerns, there are no published studies specifically investigating treatment de-escalation in pediatric patients. AQP4-Ab NMOSD is rare in children, accounting for fewer than 5% of cases.^31^ Pediatric-onset disease mirrors adult NMOSD in clinical phenotype, with a relapsing course and severe, often incomplete recovery after attacks.^32^ Given this, lifelong immunosuppressive treatment is generally recommended even in children, and discontinuation should be strongly discouraged unless severe treatment-related adverse events occur. In such cases, transitioning to an alternative agent rather than stopping treatment altogether might be advised.

In contrast, MOGAD has a strong predilection for childhood onset. Pediatric MOGAD exhibits greater phenotypic heterogeneity, a lower relapse rate (approximately 30%), and often better recovery postattack than adult disease.^33^ These characteristics support a strategy of treatment de-escalation and discontinuation in selected children.^34^ De-escalation should be personalized, considering treatment-related adverse effects, relapse severity, recovery, and factors such as MOG-IgG serostatus and clinical phenotype.

### Key Recommendation

In children with AQP4-Ab NMOSD, lifelong immunosuppression is recommended and discontinuation strongly discouraged given the severe relapsing course; in pediatric MOGAD, where relapse rates are lower and recovery generally better, cautious de-escalation and discontinuation should be considered guided by serostatus, clinical phenotype, relapse severity, and treatment tolerability.

### Late-Onset Disease

Although younger patients tend to have more inflammatory disease activity, older patients are at greater risk for disability accumulation, possibly because of reduced plasticity and repair capacity.^16^ In late-onset disease (which is usually considered to be equal or above 50 years at onset), treatment escalation to higher-efficacy therapy may not be necessary if patients present with a mild disease course and good recovery from initial attacks. However, careful consideration must be given to comorbidities and treatment-related adverse events, which are more common in older adults.

In AQP4-Ab NMOSD, late-onset patients exhibit a worse functional outcome than younger counterparts, with older age at onset independently predicting increased disability accumulation.^35^ Higher residual disability after the first attack and reduced reparative capacity with age likely contribute to this trajectory. Despite a similar relapse rate and time to first relapse compared with early-onset cases, patients with late-onset AQP4-Ab NMOSD more frequently reach significant disability thresholds (e.g., EDSS ≥6).^36^ Thus, despite frailty and comorbidities, discontinuation should not be proposed in late-onset AQP4-Ab NMOSD.

In contrast, recent data on late-onset MOGAD^37^ suggest a more benign disease trajectory with moderate relapse risk (47% at 22 months) and good recovery after attacks, with no significant difference in long-term disability compared with early-onset cases. It is important that this cohort experienced a high frequency of treatment-related adverse events, including serious infections, underscoring the importance of re-evaluating the need for prolonged immunosuppression in older adults with MOGAD.^37^ These findings support consideration of treatment de-escalation or time-limited therapy in carefully selected patients with late-onset MOGAD, particularly where biomarkers and clinical features (e.g., relapse history, MOG-IgG serostatus, MRI activity, disability level) suggest a lower relapse risk.

### Key Recommendation

In late-onset AQP4-Ab NMOSD, treatment discontinuation should not be proposed despite frailty and comorbidities, given the greater risk of disability accumulation with age; in late-onset MOGAD, where disease trajectory is more benign but treatment-related adverse events are frequent, cautious de-escalation may be considered in carefully selected patients guided by relapse history, serostatus, MRI activity, and disability level.

### Pregnancy

**Figure 1:**
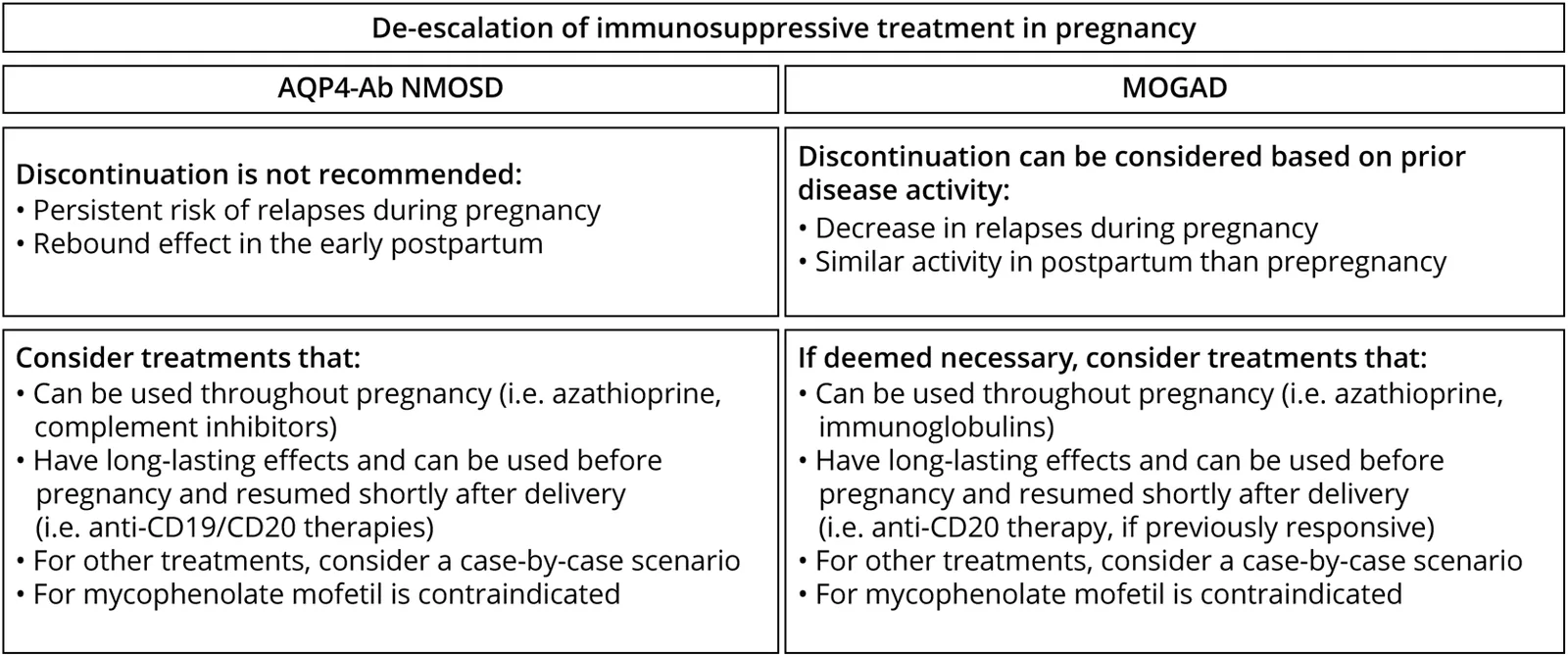
Treatment De-Escalation in NMOSD and MOGAD During Pregnancy For AQP4-Ab NMOSD, treatment maintenance is recommended during pregnancy and postpartum and thus should rely on drugs that can be used during pregnancy or have a long-lasting effect covering the pregnancy period. Some drugs are contraindicated (MMF). For MOGAD, a case-by-case scenario is proposed based on the disease activity, duration of treatment, and delay from disease onset. In any case, pregnancy should be considered during disease stability periods. **In exceptional cases, treatment can be started or continued throughout pregnancy (i.e., patients active during pregnancy). AQP4-Ab = aquaporin-4 antibody; MMF = mycophenolate mofetil; MOGAD = myelin oligodendrocyte glycoprotein antibody–associated disease; NMOSD = neuromyelitis optica spectrum disorder.

Pregnancy is a common context in which treatment de-escalation or discontinuation is considered, but approaches differ significantly between AQP4-Ab NMOSD and MOGAD. Disease-modifying treatments must be evaluated for maternal and fetal safety and disease control (Figure 1).

In AQP4-Ab NMOSD, treatment discontinuation is generally not recommended. Data from a retrospective multicenter international cohort including 89 pregnancies in 58 women (46 AQP4-Ab) demonstrated that relapse occurred in 26% of treated pregnancies compared with 53% of untreated pregnancies.^38^ In addition, a large single-center cohort from China (202 pregnancies in 112 women with NMOSD) showed that relapse risk peaks in the early postpartum period, with annualized relapse rate increasing from 0.23 prepregnancy to 1.44 in the first trimester postpartum, and that lack of immunotherapy during pregnancy/postpartum independently predicted pregnancy-related attacks.^39^ These data support avoidance of treatment withdrawal. When teratogenic therapies are stopped preconception, switching to pregnancy-compatible agents or timing anti-CD20 therapy before conception are preferred strategies.^40^ Early postpartum reinitiation of therapy is essential. De-escalation may be considered only in highly selected stable patients, with close monitoring and a predefined postpartum plan.

In MOGAD, available data support a more individualized approach. A multicenter Chinese cohort of 21 patients (28 pregnancy-related attacks) reported that 39.3% (11/28) of attacks occurred in the first trimester postpartum, with annualized relapse rate increasing from 0.40 prepregnancy to 0.60 postpartum.^41^ In contrast, a multicenter French cohort of 25 pregnancies observed no relapses during pregnancy despite treatment discontinuation in a proportion of patients, and 3 relapses within the first 3 months postpartum, with annualized relapse rate decreasing from 0.67 prepregnancy to 0 during pregnancy and 0.22 postpartum.^42^ These findings suggest that treatment discontinuation during pregnancy may be reasonable in selected patients, particularly those with a monophasic course or prolonged remission. However, patients with prior relapsing disease may benefit from continued therapy, most commonly intravenous immunoglobulin, and early postpartum treatment resumption should be considered.

Overall, de-escalation during pregnancy requires individualized risk assessment, with avoidance of treatment withdrawal in AQP4-Ab NMOSD and cautious, selective discontinuation in MOGAD, alongside proactive postpartum management (Table 1).

### Key Recommendation

In AQP4-Ab NMOSD, treatment withdrawal during pregnancy is not recommended given the high relapse risk, particularly in the early postpartum period; switching to pregnancy-compatible agents and prompt postpartum reinitiation are preferred strategies. In MOGAD, treatment discontinuation during pregnancy may be reasonable in selected patients with monophasic disease or prolonged remission, but those with relapsing disease should continue therapy. Early postpartum resumption should be planned in all cases.

## Toward Monitoring Treatment De-Escalation/Discontinuation With Imaging

Interattack asymptomatic MRI lesion accrual appears to be uncommon in both AQP4-Ab NMOSD and MOGAD,^43-45^ with available data indicating that new lesions are infrequent in the absence of clinical relapse and that radiologic activity typically reflects clinical disease activity rather than subclinical progression. In any case, clinicians should be aware of the risk of new MRI activity associated with disease reactivation, particularly within the first 12 months after treatment de-escalation or discontinuation.^11^

In MOGAD, MRI lesions associated with acute attacks often resolve, and lesion evolution is dynamic.^46^ New or evolving lesions may occur in the context of an ongoing clinical attack, reflecting a radiologic lag rather than true interattack disease activity.^47^ In a pediatric cohort of 97 patients, asymptomatic lesions beyond the initial follow-up scan were more frequently observed in those receiving maintenance immunotherapy compared with untreated patients, suggesting that treatment may suppress clinical relapses without fully abolishing inflammatory activity.^45^ Asymptomatic optic nerve enhancement has been reported in approximately 13% of follow-up scans in patients with prior optic neuritis and does not necessarily indicate active disease.^48^ Overall, the frequency and duration of MRI monitoring after treatment de-escalation in MOGAD remain uncertain.

It is important that the role of MRI after treatment de-escalation in both conditions is not to detect silent disease activity, but to identify early imaging features of clinical relapse. Accordingly, brain and spinal cord MRI should be performed approximately 3–6 months after treatment reduction, with timing tailored to the expected duration of action of the specific therapy. Subsequent imaging should be individualized based on relapse risk. Brain and spinal cord imaging provide complementary information; however, brain lesions may present with initially nonspecific symptoms, such as headache or lethargy, particularly in younger children, whereas spinal cord and optic nerve involvement are more likely to manifest with overt motor or visual deficits. In contrast, in patients with disease limited to the optic nerves, routine rebaselining MRI may be of limited value. The definition of “radiological activity” remains uncertain; thresholds derived from multiple sclerosis (e.g., one to 3 new T2 lesions) have not been validated in NMOSD or MOGAD and should be interpreted with caution.

Optical coherence tomography (OCT) may be useful to monitor patients with AQP4-Ab NMOSD and MOGAD, mainly for quantifying neuroaxonal damage after optic neuritis even when they are not on treatment. Subclinical changes in OCT may occur outside optic neuritis episodes in AQP4-Ab NMOSD,^49^ and in some cases, this is linked to contralateral involvement after unilateral optic neuritis. On the other hand, attack independent retinal neurodegeneration has not been detected in MOGAD and resolution of edema may continue for up to 12 months after optic neuritis.^50^ Rebaselining after an ON could be helpful to judge future attacks of ON.

### Key Recommendation

MRI after treatment de-escalation should be performed approximately 3–6 months after reduction, with timing tailored to the expected duration of action of the specific therapy; its purpose is to identify early imaging features of clinical relapse rather than detect subclinical activity. Subsequent imaging frequency should be individualized based on relapse risk, with particular vigilance in the first 12 months after withdrawal. MS-derived radiologic activity thresholds have not been validated in NMOSD or MOGAD and should not be directly applied. OCT provides complementary monitoring in patients with prior optic neuritis, particularly in AQP4-Ab NMOSD where subclinical retinal changes may occur between attacks.

## Toward Monitoring Treatment De-Escalation/Discontinuation With Fluid Biomarkers

Fluid biomarkers represent potential valuable predictive tools for assessing disease evolution and assessing therapeutic efficacy. Table 2 summaries key fluid biomarkers. However, although they may assist in identifying patients with unfavorable clinical trajectories, their utility in guiding treatment de-escalation in NMOSD and MOGAD is limited. The prognostic value of antibody titers in AQP4-Ab NMOSD and MOGAD remains controversial. At disease onset, titers have not reliably predicted relapse risk in MOGAD,^51^ but recent data indicate that higher MOG-IgG titers measured during remission (>1 months postevent) are associated with future relapse risk.^52^ Although not used clinically to guide treatment decisions in MOGAD, sustained low titers (i.e., near assay cutoff or declining titers without seroconversion) or seroconversion (MOG-Ab are negative) in stable patients over 4–5 years could support treatment de-escalation.^33^ In addition, cutoff positivity across different clinical and research laboratory varies and titers can decline by up to 40% over time.^52^ In AQP4-Ab NMOSD, early studies proposed antibody titers as markers of therapeutic response, but subsequent clinical trials (particularly involving anti-CD19 therapies) have demonstrated limited utility.^53^

**Table 2:** Body Fluid Biomarkers and Their Potential Utility to Monitor Disease Activity and De-Escalation Strategies in NMOSD and MOGAD

Biomarker	Disease	Findings	Conclusion
Antibody titres	NMOSD/MOGAD	• Conflicting evidence on baseline titers predicting relapse.^51^ • In MOGAD, longitudinal antibody dynamics may predict group-level but not individual relapse risk.^52^ • AQP4-Ab titers do not correlate with treatment response (e.g., anti-CD19).^53^	Antibody titer itself is not reliable for monitoring disease activity or guiding treatment de-escalation Seroconversion of MOG-IgG can be useful for general discontinuation consideration
B-cell subsets	NMOSD	• CD20^+^ B cell counts at 6 mo may predict future relapse risk and MRI activity • CD27^+^ memory B-cell monitoring can optimize rituximab dosing • Subsets (CD20, CD27, plasmablasts) may reflect biological response to B-cell–depleting therapy^10^	Useful for monitoring treatment response and currently used to guide de-escalation
	MOGAD	• Patients may relapse despite complete CD20^+^ B-cell depletion, suggesting distinct pathophysiology.^10^	B-cell counts do not predict treatment response or guide de-escalation in MOGAD.
Biomarkers of neuronal and glial injury (sGFAP, sNfL)	NMOSD	• sGFAP predicts relapse and increases with subclinical activity^5456^ • sNfL correlates with disability during attacks.^54^ • Levels decline with anti-CD19/complement therapies	Astroglial and axonal biomarkers may reflect disease activity and treatment response but are not validated for de-escalation guidance
	MOGAD	• sNfL and sGFAP correlate with disability.^55^ • sNfL may predict future relapses.^55^	Promising for disease monitoring; their role in guiding de-escalation requires further study
Cytokines/chemokines	NMOSD/MOGAD	• Elevated Th17 (IL-6, IL-8, GM-CSF), Th1 (IFN-γ), and Treg (IL-10) responses during attacks.^56^ • Multiple cytokines linked to NMOSD (e.g., IL-17A, BAFF, CXCLs) • BAFF may have predictive value in MOGAD.^56^	Although immunologically relevant, cytokines/chemokines are not yet robust biomarkers for treatment monitoring or de-escalation
Complement factors	NMOSD	• C3, C4, C5b-9 elevated during attacks^57^ • Inconsistent findings during remission • sC3a correlates with disability • CH50 reduced with eculizumab	Interpretation limited by small studies and methodological variability; not reliable for monitoring or de-escalation
	MOGAD	• CSF C4a and C4a/C4 ratio linked to relapse.^58^ • CSF SC5b-9 associated with EDSS ≥3 • Elevated serum C5a, SC5b, C3a, Bb^60^	Similar limitations apply; current evidence does not support use in guiding treatment de-escalation

Abbreviations: AQP4-Ab = aquaporin-4 antibody; BAFF = B-cell activating factor; CXCL = C-X-C motif chemokine ligand; MOGAD = myelin oligodendrocyte glycoprotein antibody–associated disease; MOG-IgG = myelin oligodendrocyte glycoprotein immunoglobulin G; NMOSD = neuromyelitis optica spectrum disorder; sGFAP = serum glial fibrillary acidic protein; sNfL = serum neurofilament light chain.

B-cell subsets after B-cell–depleting therapies may have potential utility as markers of treatment response. Notably, lower B-cell counts at 6 months postinfusion were associated with reduced disease activity, including fewer relapses and new T2 brain MRI lesions at 2 years. Thus, monitoring B-cell subsets may help to inform treatment optimization, although their role in guiding de-escalation strategies in NMOSD remains uncertain. Conversely, in MOGAD, B-cell counts are not reliable indicators of treatment efficacy, as relapses may occur despite undetectable B-cell levels during depletion therapy.^10^ High-sensitivity assays for biomarkers of neuronal and glial injury have enabled serum-based monitoring of disease activity. In NMOSD, data from the N-MOmentum trial demonstrated that serum neurofilament light chain (sNfL) strongly correlated with EDSS worsening during attacks and predicted postattack disability.^54^ Although baseline glial fibrillary acidic protein (GFAP) levels were reported to be associated with relapse risk, these measurements were obtained relatively soon after preceding attacks in many cases and may, therefore, reflect residual effects of prior disease activity rather than true predictive value. Consistent with this, studies measuring sGFAP and sNfL during remission (≥6 months postattack) have not demonstrated an association with subsequent relapse risk, whereas higher EDSS scores were associated with increased likelihood of early relapse.^59^ Both GFAP and NfL remained lower in attack-free treated individuals compared with placebo, and in relapsing patients, which highlight their potential as markers of disease activity rather than predictors of relapse.^54,60^ In addition, biomarker dynamics over time may be more informative than single time point measurements, and the ability to detect an imminent relapse based on isolated GFAP or NfL levels remains uncertain.

In MOGAD, baseline NfL levels have emerged as a potential marker of disease activity.^55^ Their interpretation is influenced by age, sex, and BMI and, therefore, requires normalization (e.g., z-scores) to account for these factors. Although this improves comparability across individuals, it may limit clinical utility at the individual level, particularly in the absence of longitudinal measurements.

Other biomarkers such as cytokines/chemokines or complement obtained both in serum or CSF have been studied in both NMOSD and MOGAD,^56-58^ with conflicting results, likely because of the high variability of these molecules over the disease course and differences in assay methodologies across studies.

### Key Recommendation

No fluid biomarker is currently sufficiently validated to guide treatment de-escalation decisions in isolation; in MOGAD, sustained low MOG-IgG titers or seroconversion over 4 to 5 years may support de-escalation in otherwise stable patients, but biomarkers should be interpreted alongside clinical and radiologic assessment rather than used as standalone decision-making tools.

## Equity and Global Health, Health Economic, and Patient Perspectives

Many patients seek clearer guidance on treatment de-escalation, weighing reduced immunosuppression against relapse risk. De-escalation decisions must be grounded in robust evidence, delivered through shared decision making, and aligned with individual patient goals across physical, psychological, and social dimensions. The high cost of maintenance therapies, particularly complement inhibitors, inebilizumab, and satralizumab in AQP4-Ab NMOSD, raises sustainability concerns. Identifying patients in whom therapy can be safely tapered or discontinued could yield substantial savings at both the system and individual level. Globally, access to diagnostics and therapeutics remains inequitable; in low- and middle-income countries, de-escalation may be driven by resource constraints rather than clinical indication, where the absence of reliable monitoring or rescue therapy amplifies associated risks. Future research must incorporate patient-reported outcomes and diverse populations and health care settings to ensure equitable, generalizable, and patient-centered treatment frameworks.

## Conclusion and Future Directions

**Figure 2:**
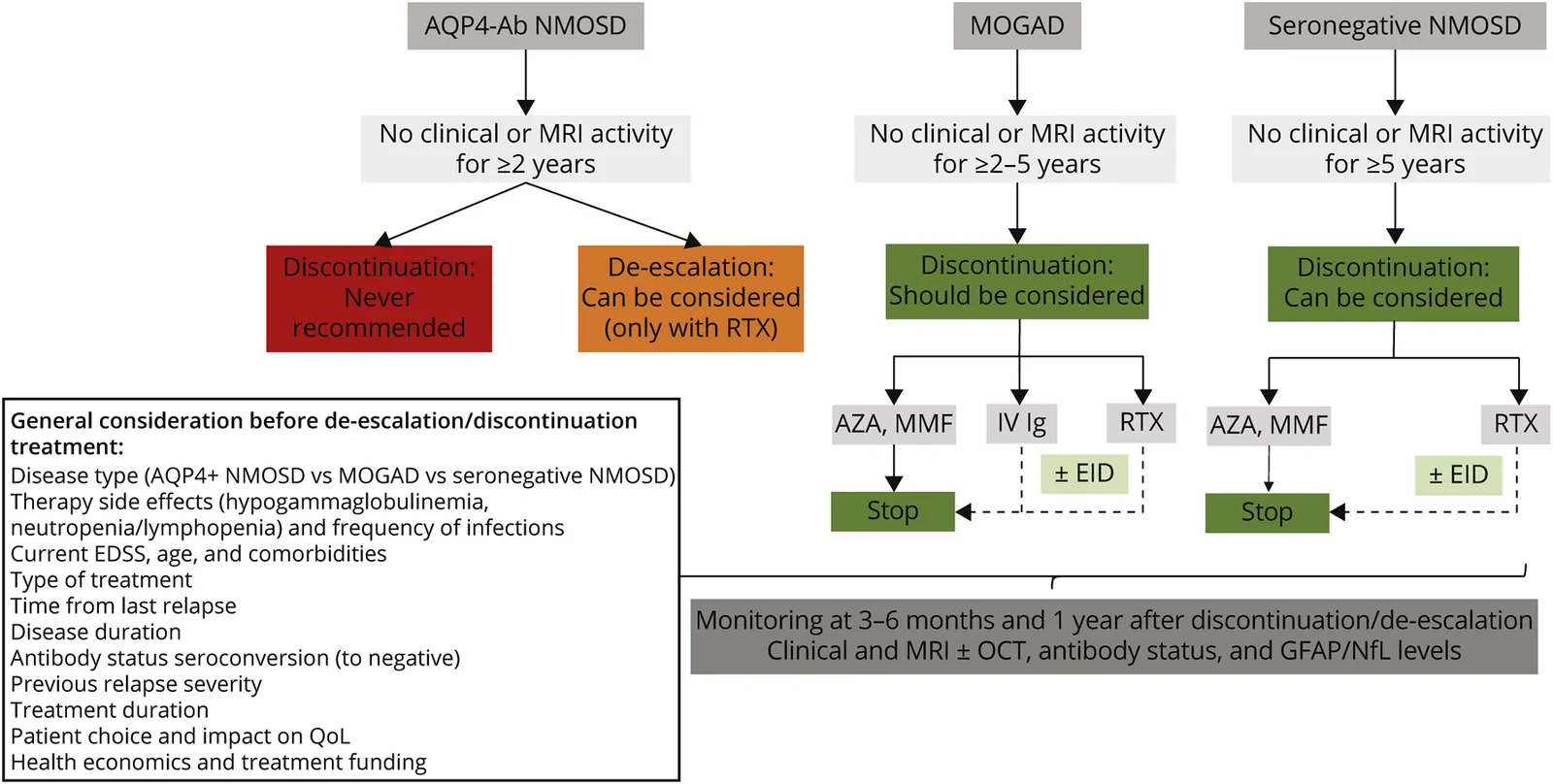
Key Considerations and Strategies for Treatment De-Escalation/Discontinuation in AQP4-Ab NMOSD and MOGAD This schematic outlines the clinical, biological, and practical factors guiding treatment de-escalation/discontinuation. Key considerations before de-escalation include antibody status, relapse history, severity and recovery, current disability (EDSS), comorbidities, treatment type and duration, side effects, infection risk, and patient preferences. In AQP4-Ab NMOSD, because of high relapse risk, de-escalation may be considered only in stable patients with emerging evidence supporting anti-CD20 therapies as a favorable platform. In MOGAD, where monophasic disease is more common, discontinuation may be considered after 2–5 years in children and adults without relapses. De-escalation in MOGAD may be considered in stable patients treated with anti-CD20 or IVIG. In seronegative NMOSD, treatment discontinuation could be considered after 5 years without disease activity. Monitoring may include MRI, OCT, and fluid biomarkers around the anticipated time of therapeutic waning and at follow-up intervals. AQP4-Ab = aquaporin-4 antibody; AZA = azathioprine; EDSS = Expanded Disability Status Scale; EID = extended interval dosing; GFAP = glial fibrillary acidic protein; IVIg = intravenous immunoglobulin; MMF = mycophenolate mofetil; MOGAD = myelin oligodendrocyte glycoprotein antibody–associated disease; Nfl = neurofilament light chain; NMOSD = neuromyelitis optica spectrum disorder; OCT = optical coherence tomography; RTX = rituximab.

Treatment de-escalation and/or discontinuation in AQP4-Ab NMOSD and MOGAD are increasingly relevant in long-term disease management, driven by improved relapse control, treatment toxicity, patient preference, and health-economic considerations. However, the evidence base remains limited and largely retrospective, and decisions must remain individualized. In AQP4-Ab NMOSD, current evidence supports continuation of maintenance immunotherapy in all patients. Relapses are often severe and disabling, and observational data demonstrate a high risk of disease reactivation after treatment withdrawal. Discontinuation is, therefore, not recommended. De-escalation may be considered only in selected patients with prolonged clinical and radiologic stability, low relapse burden, and significant risk of treatment-related toxicity or comorbidity. Even in this setting, relapse risk persists and requires close monitoring. Where treatment reduction is considered, it is based on interval extension or dose reduction with B-cell–depleting therapies. In MOGAD, disease course is more heterogeneous and may allow treatment discontinuation in selected patients. Relapse risk appears to decline over time, particularly in patient with a single clinical attack. Discontinuation may be considered after sustained remission, typically after 2–5 years in children and in adults without clinical or radiologic disease activity. Longer treatment exposure before withdrawal appears protective. Patients with a single clinical attack, more than 2 years of treatment exposure, and subsequent seroconversion to MOG-IgG negativity appear at lower risk of relapse. In contrast, patients with prior relapsing disease, shorter remission duration, or severe attacks require a more cautious approach. Treatment-specific factors are important. Oral immunosuppressants such as azathioprine and mycophenolate mofetil are usually stopped rather than tapered. B-cell depletion therapies and IVIg may be de-escalated through infusion interval extension or dose reduction before cessation. For other drugs (anti-IL6R, complement inhibitors), data on optimization strategies are still very limited. In any case, a clear relapse contingency plans and rapid access to treatment re-initiation should be in place. In double seronegative NMOSD, evidence guiding discontinuation is sparse. In patients with prolonged remission, generally beyond 5 years, discontinuation may be considered, particularly in monophasic disease, but recommendations remain tentative. Monitoring after treatment reduction is essential but not standardized. Clinical review and MRI surveillance are recommended around the anticipated period of therapeutic waning, typically within 3–6 months after de-escalation and again at 12 months, with continued follow-up for at least 2 years. OCT and fluid biomarkers such as GFAP and neurofilament light chain may provide supportive information, but no biomarker has been validated to predict relapse after treatment withdrawal. Recommendations are summarized in Figure 2. Future work should prioritize prospective registries capturing treatment duration, de-escalation strategies, and relapse outcomes. Harmonized datasets integrating imaging, serology, and patient-reported outcomes are needed to refine risk stratification. Registry-embedded and platform discontinuation trials in carefully selected stable populations will be essential to generate higher-quality evidence. Mechanistic studies examining antibody dynamics, B-cell repopulation, and glial injury biomarkers may further inform individualized decision making. In summary, maintenance immunotherapy remains the standard approach in AQP4-Ab NMOSD, where discontinuation is generally not advised. In MOGAD, cautious and individualized withdrawal may be feasible after sustained remission, particularly in monophasic or in those that become seronegative patients. Until prospective evidence is available, de-escalation decisions should be guided by disease course, relapse history, treatment duration, disability, and structured postwithdrawal monitoring.

## Glossary

AQP4-Ab: aquaporin-4 antibody
DMT: disease-modifying therapy
MOGAD: myelin oligodendrocyte glycoprotein antibody–associated disease
MOG-IgG: myelin oligodendrocyte glycoprotein immunoglobulin G
NMOSD: neuromyelitis optica spectrum disorder
